# Promitotic Action of *Oenothera biennis* on Senescent Human Dermal Fibroblasts

**DOI:** 10.3390/ijms232315153

**Published:** 2022-12-02

**Authors:** Sara Ceccacci, Kévin Roger, Ines Metatla, Cerina Chhuon, Khaled Tighanimine, Stefano Fumagalli, Adriana De Lucia, Iwona Pranke, Corinne Cordier, Maria Chiara Monti, Ida Chiara Guerrera

**Affiliations:** 1 Department of Pharmacy, University of Salerno, 84084 Fisciano, Italy; 2 PhD Program in Drug Discovery and Development, University of Salerno, 84084 Fisciano, Italy; 3 Proteomics Platform Necker, Université Paris Cité-Structure Fédérative de Recherche Necker, INSERM US24/CNRS UAR3633, 75015 Paris, France; 4 Institut Necker Enfants Malades, INSERM U1151, 75015 Paris, France; 5 Arterra Bioscience SpA, 80142 Naples, Italy; 6 Cytometry Platform, Structure Fédérative de Recherche Necker, INSERM US24-CNRS UAR3633, 75015 Paris, France

**Keywords:** *Oenothera biennis* hydrophilic extract, senescence, fibroblasts, proteomics, mass spectrometry, diaPASEF, mitosis, skin aging

## Abstract

Accumulation of senescent dermal fibroblasts drives skin aging. The reactivation of proliferation is one strategy to modulate cell senescence. Recently, we reported the exact chemical composition of the hydrophilic extract of *Oenothera biennis* cell cultures (ObHEx) and we showed its skin anti-aging properties. The aim of this work is to assess its biological effect specifically on cell senescence. ObHEx action has been evaluated on normal human dermal fibroblasts subjected to stress-induced premature senescence (SIPS) through an ultra-deep proteomic analysis, leading to the most global senescence-associated proteome so far. Mass spectrometry data show that the treatment with ObHEx re-establishes levels of crucial mitotic proteins, strongly downregulated in senescent cells. To validate our proteomics findings, we proved that ObHEx can, in part, restore the activity of ‘senescence-associated-ß-galactosidase’, the most common hallmark of senescent cells. Furthermore, to assess if the upregulation of mitotic protein levels translates into a cell cycle re-entry, FACS experiments have been carried out, demonstrating a small but significative reactivation of senescent cell proliferation by ObHEx. In conclusion, the deep senescence-associated global proteome profiling published here provides a panel of hundreds of proteins deregulated by SIPS that can be used by the community to further understand senescence and the effect of new potential modulators. Moreover, proteomics analysis pointed to a specific promitotic effect of ObHEx on senescent cells. Thus, we suggest ObHEx as a powerful adjuvant against senescence associated with skin aging.

## 1. Introduction

Senescence is the main cause of ageing and age-related diseases. Senescent cells undergo proliferation arrest as a response to physiological telomere erosion (replicative senescence) or to stress triggers including DNA damage, oxidative stress, organelle strain and oncogene activation (stress induced premature senescence, SIPS) [1]. Despite DNA Damage Response (DDR) induced growth arrest, senescent cells display a high metabolic state and lysosomal activity, crucial for their increased secretory activity, known as senescence-associated secretory phenotype (SASP) [2,3]. SASP factors include several families of soluble (interleukins, chemokines, growth factors, secreted proteases) and insoluble (secreted insoluble proteins and extracellular matrix components) proteins that negatively affect the surrounding tissue, generating a chronic low-grade inflammatory state.

The accumulation of senescent fibroblasts in derma drives skin aging, impairing the integrity of the extracellular matrix (ECM) and the function of neighbouring microvascular endothelial cells, epidermal melanocytes and keratinocytes [4,5,6]. The three main strategies to counteract senescence include: (i) the clearance of senescent cells by the induction of apoptosis or immune activation; (ii) the modulation of the SASP and (iii) the reactivation of cell proliferation. Indeed, although senescence has been originally described as an irreversible cell cycle arrest, current evidence suggests that proliferation can be restored [7,8,9].

In this scenario, ingredients able to prevent and/or modulate cellular senescence are of great interest in the cosmetic field as valuable weapons to counteract skin aging. In particular, extracts derived from the species *Oenothera biennis* (*O. biennis*, Evening Primrose), belonging to the family *Onagraceae*, are traditionally used for cosmetic applications due to their anti-inflammatory and antioxidant activities [10]. Methanolic extracts prepared from aerial parts and aqueous leaf extracts mainly contain phenolic acids and flavonoids, whereas methanolic extracts obtained from roots mostly contain sterols and triterpenes [11]. Although the biological activities of alcoholic and hydroalcoholic aerial part extracts have been demonstrated [12,13,14], the most frequently used extracts of *O. biennis* are the oils obtained from seeds, which are extremely rich in fatty acids. Indeed, since their high content of linoleic acid (LA) and γ-linolenic acid (GLA), *O. biennis* seed oils improve the proper functioning of the skin and, in particular, they have showed beneficial effects in patients with chronic dermatitis [11,15].

More recently, extracts obtained from *O. biennis* cell cultures have been proposed to fully substitute extracts from spontaneous plants as they are bio-sustainable, contaminant-free and standardized secondary metabolites mixtures [16]. It is precisely the combination of the compounds with their synergistic effect that is responsible for the overall activity of botanical extracts [17].

Here, we have focused on the hydrophilic extract of *O. biennis* cell cultures (ObHEx), rich in lignans and triterpenes, as it showed skin anti-aging properties when tested in in vitro and ex vivo models [18]. Mass spectrometry based chemical characterization of this extract revealed that it contains several classes of interesting secondary metabolites. They belong to lignans (salvadoraside and liriodendrin) and triterpenes (myrianthic acid, arjunolic acid, asiatic acid and hederagenin), some of which have previously been associated with pro-collagen I production in human fibroblasts [19,20]. In particular, we have previously proved that ObHEx promotes matrix collagen contraction, actin polymerization and the production of ECM proteins, thus improving skin firmness and elasticity, via an increase in Myosin light chain kinase (MYLK) gene expression.

**Figure 1:**
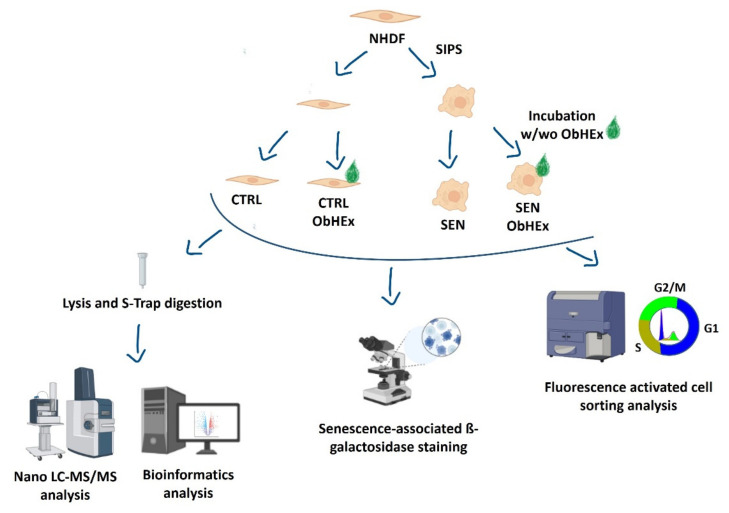
Summary scheme (created with BioRender.com accessed on 12 November 2022) of the adopted methodology.

To date, the molecular mechanisms of ObHEx effects on skin aging and on cellular senescence, in particular, have not been investigated. The aim of this work is to explore ObHEx activity on senescent human dermal fibroblasts. We used a data-independent mass spectrometry ultra-deep proteomic approach to obtain hints of the mechanism of action and bio-orthogonal methods to validate and investigate it (Figure 1).

## 2. Results

Stress-induced premature senescence (SIPS) was performed in normal human dermal fibroblasts (NHDF) by treatment with hydrogen peroxide (H_2_O_2_). Oxidative stress-induced senescent cells are an excellent in vitro model for aging research and H_2_O_2_ is widely used to achieve it [21,22].

To understand the biological pathways altered by ObHEx on senescent NHDF cells, we used an ultra-deep proteomic approach. SIPS and control cells were treated or not for 48 h with the extract at 0.01% (*p*/*v*) since, from the MTT assay reported in [18], this results in the highest concentration that does not cause any cell toxicity after 48 h incubation.

Cell lysates were then subjected to tryptic digestion and nano-UPLC MS/MS analysis. In order to gain the optimal depth of the proteome, mass spectrometry data were acquired in Data Independent Acquisition (DIA) mode, which allowed the robust quantification of 9650 proteins (Table S1), with a data completeness of 98%. Our study reports the most complete proteome analysis of senescent cells to date.

### 2.1. Stress-Induced Premature Senescence (SIPS) Evaluation

First of all, we verified if the senescence induction in NHDF was successful, analysing the global proteome of H_2_O_2_ treated NHDF by mass spectrometry (MS) and examining their morphology by flow cytometry.

#### 2.1.1. SIPS Evaluation by Mass Spectrometry (MS)

**Figure 2:**
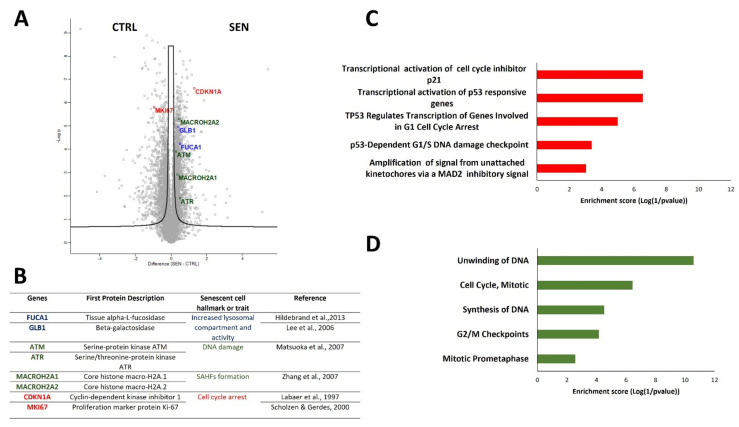
(**A**) Volcano plot representing the statistical comparison of the protein LFQ intensities of H_2_O_2_ treated cells versus untreated controls. The abscissa reports the fold change in logarithmic scale (difference), and the ordinate −log(p) of *t*-tests (FDR = 0.05, s0 = 0.1). Deregulated senescent protein markers have been highlighted in different colours in the volcano plot and described in detail in the related table [23,24,26,27,28,29] (**B**). (**C**) Reactome enrichment analysis of the most upregulated and downregulated (**D**) proteins (FDR = 0.05, s0 = 1) in H_2_O_2_ treated cells. In both cases, only the main five pathways have been reported (*p* < 0.01).

To evaluate SIPS induction, we compared the total proteome of H_2_O_2_ treated NHDF cells versus untreated ones. Among the 3256 proteins that were deregulated, several biomarkers of senescence were found to be significantly altered (Figure 2A,B). Firstly, we observed the upregulation of the most widely used biomarker of senescent cells, the ‘senescence-associated-ß-galactosidase’ (SA-β-gal, GLB1). The increased levels of the lysosomal enzymes GLB1 and Tissue alpha-L-fucosidase (FUCA1), which has also been found upregulated by MS, reflect the characteristic increment in lysosomal content of senescent cells [23,24]. Indeed, lysosomes favour catabolic processes, which provide energy and raw materials required by senescence associated secretion [25].

Secondly, the protein level of serine/threonine-protein kinase ATR and serine-protein kinase ATM, both involved in DDR signalling, was also found to be increased [26]. DDR is activated by nuclear DNA damage, generally associated with the establishment of cellular senescence, and it converges into the activation of tumour suppressor p53. In addition to this, the upregulation of the histone 2A variants MACROH2A1 and MACRO2A2 has also been observed. These proteins participate in the ATR dependent formation of Senescence Associated Heterochromatin Foci (SAHFs), dense and repressive chromatin structures characteristic of senescent cells [1,2,27].

Lastly, the deregulated levels of Cyclin Dependent Kinase Inhibitor 1A or p21 (CDK1NA) and proliferation marker protein Ki-67 (MKI67) reflect senescent cell cycle arrest at the G2 phase [28,29]. Indeed, p21 increases levels, promoted by a DNA damage-induced p53 pathway, and inhibits the CDK2-cyclin E complex. This causes the dephosphorylation of retinoblastoma protein (RB), which in its hypo-phosphorylated state sequesters E2F, a transcriptional factor that favours the progression of the cell cycle [2].

Beyond these already known markers of senescence, a broader pathway enrichment analysis of the most upregulated proteins in H_2_O_2_ treated versus control cells points to p53/p21 pathway activation induced by DNA damage (Figure 2C, Table S2). Instead, proteins associated with proliferation and mitosis are downregulated, pointing to the consequent proliferation arrest (Figure 2D, Table S3).

#### 2.1.2. SIPS Evaluation by Flow Cytometry

SIPS has also been confirmed by flow cytometry analysis. We observed an increased in cytoplasmic granularity of H_2_O_2_ treated cells (Figure S1A,B), due to the augmentation in number and size of the lysosomes in senescent cells. This is the result of the balance between the gradual accumulation of dysfunctional lysosomes and the production of new ones [25]. Moreover, we also detected an intra-lysosomal accumulation of lipofuscin aggresomes: this growth, associated with an increment of autofluorescence in the 525/50 nm bandpass channel of the flow cytometer, is another marker of senescence related to lysosomal malfunction (Figure S1C,D) [30].

### 2.2. ObHEx Partially Restored Mitotic Proteins Downregulated in Senescent NHDF Cells

**Figure 3:**
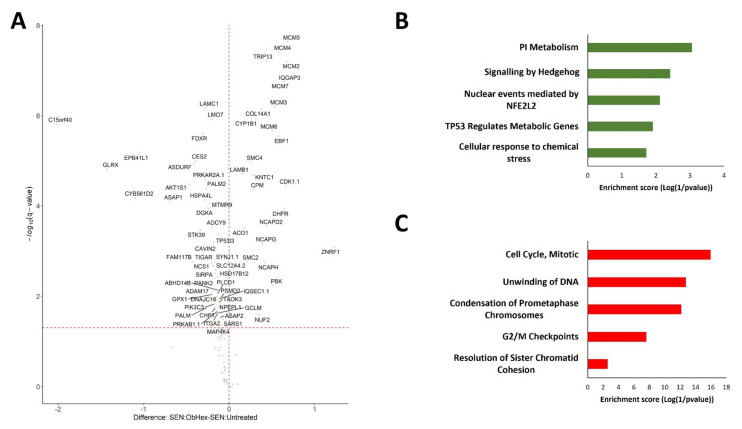
(**A**) Plot representing the statistical difference between senescent cells with the presence or absence of ObHEx extract resulting from Tukey HSD post-hoc test of Two-way ANOVA interaction factors (i.e., Induction and Treatment). The abscissa reports the fold change in logarithmic scale (difference), and the ordinate −log (*q* value) of the Tukey HSD tests for the specific SEN:ObHex/SEN:Untreated pairwise comparison while keeping the family-wise error rate low (family-wise confidence level of 0.95). (**B**) Reactome enrichment analysis of downregulated and (**C**) upregulated proteins in senescent NHDF cells after ObHEx treatment. Only the main five pathways have been reported (*p* < 0.05).

ObHEx treatment was able to partially restore the levels of 71 proteins deregulated in the senescent condition (Two-way ANOVA test *q*-value < 0.05). More precisely, 46 proteins were downregulated by the extract and 25 were upregulated (Figure 3A, Tables S4 and S5).

Reactome enrichment analysis of the 46 proteins whose levels were decreased by ObHEx suggests that the extract can reduce p53 pathway activation and affect phosphatidylinositol metabolism and Hedgehog signalling, whose alterations were found in aging [31,32] (Figure 3B, Table S6).

Regarding the 25 upregulated proteins, the enrichment analysis clearly points to mitotic cell cycle pathway reactivation and, in particular, to DNA unwinding and replication, chromosome condensation, and the resolution of sister chromatids (Figure 3C, Table S7).

**Figure 4:**
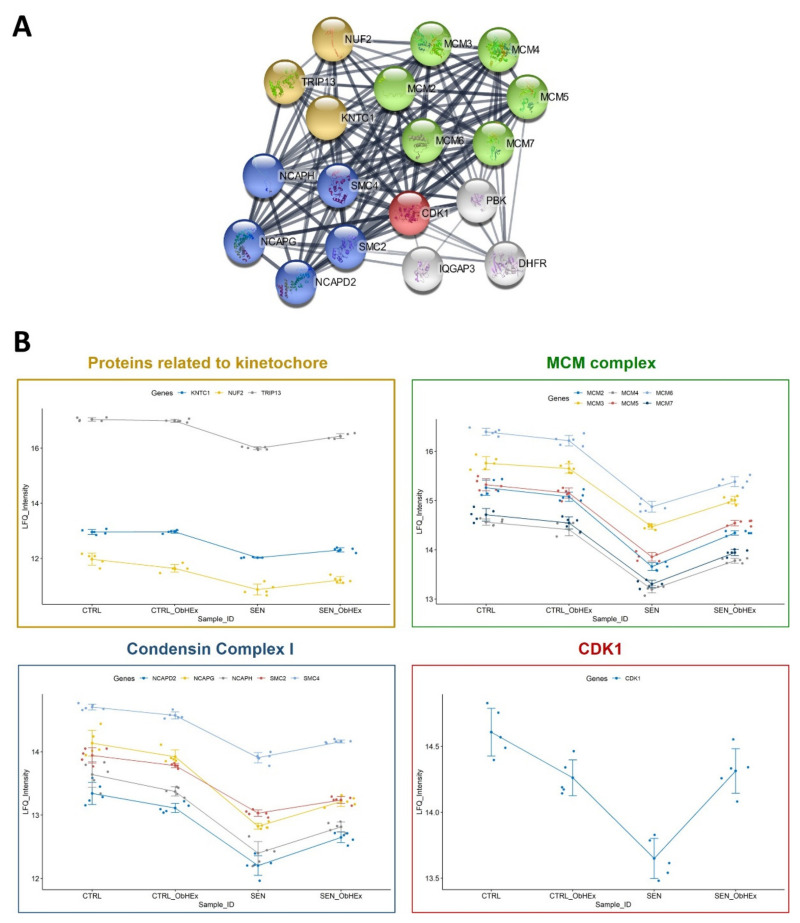
(**A**) Cluster analysis of upregulated proteins in senescent NHDF cells after ObHEx treatment. It was performed using the Cytoscape StringApp; setting 0.9 as the threshold (highest confidence). (**B**) Profile plots of the proteins included in the clusters reported in Panel (**A**). Each plot shows the mean LFQ intensity with error bars representing the error limits defined by the standard deviation.

In depth analysis shows that 18 of the 25 restored proteins cluster strongly together (Figure 4A). In this cluster, it is possible to discern CDK1, all the subunits of the condensin I complex (SMC2, SMC4, NCAPD2, NCAPH and NCAPG), three kinetochore related proteins (KNTC1, NUF2 and TRIP13), all the six subunits of the replicative minichromosome maintenance (MCM) complex (MCM2-7) together with IQGAP3, PBK and DHFR.

The ability of ObHEx to partially restore the levels of these proteins is shown well in the profile plots in Figure 4B: protein expression levels were mostly unaltered in the proliferating cells treated or not with the extract, dropped in the senescent cells, and partially increased after ObHEx treatment.

### 2.3. Biological Assays Confirmed the ObHEx Promitotic Mechanism of Action

**Figure 5:**
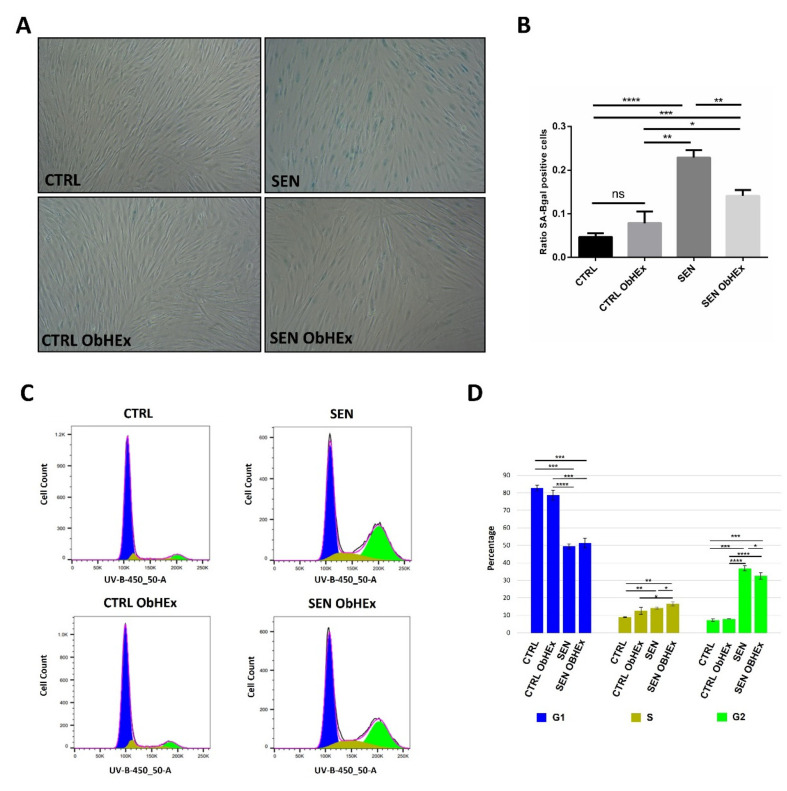
(**A**) Images (40× magnification) of the control and senescent NHDF cells treated or not with ObHEx after the senescence-associated ß-galactosidase (SA-ß-gal) assay. Positive cells are blue. (**B**) Bar graph showing the ability of ObHEx to reduce SA-ß-gal activity. (**C**) DNA profiles of proliferating and senescent fibroblasts after treatment with ObHEx or not, and (**D**) related bar graph. The fluorescence intensity of Hoechst 33342 was measured by flow cytometry with a BD LSRFortessa Cell Analyzer using UV laser (355 nm) associated to a 450/50 filter set. G1, S and G2/M phases are reported in blue, brown and green, respectively. In each bar graph, the bars represent the standard deviations while asterisks indicate significant variations (* *p* < 0.05; ** *p* < 0.01; *** *p* < 0.001, **** *p* < 0.0001) according to Student’s *t*-test. ns = not statically significant.

The most common hallmark of senescent cells is the increased SA-β-gal activity that can be evaluated by a staining assay. Our results show that H_2_O_2_ treatment induced a significant increase in the number of SA-β-gal positive cells (blue), from 4.7% to 22.9% (Figure 5A,B). ObHEx incubation significantly reduced SA-β-gal activity with a decrease of 38.4% (*p* < 0.01).

Moreover, to assess if the restoration of mitotic protein expression by ObHEx translates to the reactivation of the cell cycle, the control and senescent cells were treated or not with the extract for 72 h and Fluorescence Activated Cell Sorting (FACS) experiments were performed. The senescent cell results showed a block in the G2/M phases of the cell cycle (36.8%), while only a much lower fraction of the control cells (7.2%) were in these phases since they reached confluence, accordingly to the literature [33] (Figure 5C,D). The treatment of senescent cells with ObHEx was able to reduce the G2/M population by 4.3% (*p* < 0.05). Of notice, when related to the senescence cell portion after SIPS (22.9%), the G2/M escape could be estimated to be 18.9%. The same ObHEx treatment for 48 h has been carried out, but the results were not statistically significant.

## 3. Discussion

In this study, we investigated the effect of a hydrophilic *Oenothera biennis* cell extract (ObHEx) on cellular senescence, since it showed skin anti-aging properties when tested in in vitro and ex vivo models [18]. We used a similar senescent model of NHDF subjected to SIPS for treatment with H_2_O_2_ [21,22].

To understand the mechanism of action of ObHEx on senescent human dermal fibroblasts, by the disclosure of the biological pathways altered by the extract, we performed a data-independent mass spectrometry ultra-deep proteomic approach. It allowed us to obtain the most complete proteome analysis of senescent cells to date and, for the first time, the simultaneous quantification of numerous senescence markers.

First of all, to assess the senescence induction by H_2_O_2_ treatment on NHFD cells, we quantified the known senescence markers. Our proteomics data confirmed senescence induction by oxidative stress: indeed, we observed altered levels of already known senescence markers related to the increased lysosomal content (GLB1 and FUCA1), DNA damage (ATR, ATM, MACROH2A1 and MACRO2A2) and G2 phase cell cycle arrest (CDK1NA and MKI67). Moreover, the pathway enrichment analysis of the most downregulated proteins in H_2_O_2_ treated versus control cells points to mitosis, reflecting the senescent proliferation arrest.

Comparing the proteome of SIPS NHDF cells treated with ObHEx versus untreated ones, we found that the treatment with the extract was able to partially restore the levels of proteins and complexes playing crucial roles in several stages of mitosis. In fact, ObHEx incubation increased the levels of CDK1, a key mitotic protein that triggers the entry into mitosis by forming a complex with cyclin B [34]. Moreover, all the five subunits of the condensin I complex were upregulated. This complex is composed of two structural maintenance of chromosomes (SMC) subunits, SMC2 and SMC4, and three non-SMC subunits, NCAPD2, NCAPH and NCAPG. In the prometaphase, the function of the condensin I complex is to promote the hypercondensation of chromosomes by the introduction of positive supercoils into the DNA in an ATP-dependent manner [35]. In addition to this, the extract upregulated KNTC1, NUF2 and TRIP13, which are three proteins associated with the kinetochore, a large complex that, during the prometaphase, connects centromeric chromatin to microtubules from opposite spindle poles to favour the segregation of sister chromatids [36,37]. A partial restoration of the levels of MCM proteins has also been detected. These proteins are at the core of the replication helicase complex that unwinds double stranded DNA to provide single strands as templates for DNA polymerase. The MCM complex is converted into an active helicase during the S phase, but is already loaded onto chromatin during the telophase [38,39]. The extract incremented the levels of IQGAP3, PBK and DHFR, too. The first, IQGAP3, is an important regulator of mitotic progression because it promotes cdk7 activity, essential for Cdc2 activation [40,41]; PBK is a kinase active only in mitosis; when phosphorylated, it interacts with p53, destabilizing it and attenuating the DNA damage pathway [42]; and DHFR is a key enzyme in DNA biosynthesis whose levels are markedly attenuated in senescent human fibroblasts [43].

Bio-orthogonal assays also showed the ability of ObHEx to partially revert senescence hallmarks, namely lysosomal activity and cell cycle arrest. Indeed, to verify if the restoration of mitotic protein expression by ObHEx translates to the reactivation of the cell cycle, we performed FACS (Fluorescence Activated Cell Sorting) experiments on SIPS NHDF cells treated or not with the extract. They showed that ObHEx was able to reduce the fraction of cells blocked in the G2 phase and to promote their re-entry into the cell cycle of senescent cells.

## 4. Materials and Methods

### 4.1. Cell Culture

Normal Human Dermal Fibroblasts (NHDF; Promocell) were cultured in Dulbecco’s Modified Eagle Medium (DMEM; Gibco) supplemented with 10% of foetal bovine serum (FBS; Gibco) and 500 U/mL of penicillin-streptomycin (Gibco) in 95% air, 5% CO_2_ and a humidified atmosphere at 37 °C.

### 4.2. Induction of Stress Induced Premature Senescence (SIPS)

A total of 1′120′000 NHDF cells were seeded into each of 60 mm cell culture dishes, one day prior to their incubation with 100 µM H_2_O_2_ at 37 °C for 2 h [21,22]. Subsequently, the H_2_O_2_ was washed with Phosphate Buffered Saline (PBS; Gibco) to terminate the treatment and the cells were grown in a normal medium for 4 days. For the non-senescent cells, used as a control, 224′000 NHDF cells were seeded into each of 60 mm cell culture dishes. The experiment was performed in 5 biological replicates.

### 4.3. Oenothera Biennis Hydrophilic Extract (ObHEx) Preparation

The extract was prepared in the laboratories of Arterra Bioscience SpA [18]. The cell cultures were obtained from the leaves of *Oenothera biennis* plants (provided by GEEL Floricultura s.s) by inducing the proliferation of meristematic cells on solid agar plates until the obtained calluses. The cells were transferred to the liquid growth medium (Gamborg B5, supplemented with 2,4 dichlorophenoxyacetic acid (1 mg/L), adenine (1 mg/L), and kinetine (0.01 mg/L)) and grown as suspension cultures under orbital shaking. Once cultures of about 150 g/L were obtained, the cells were collected and lysed in PBS at pH 7.4 to prepare a water-soluble extract. After lyophilization, the obtained powder was dissolved in water or cell culture media at the appropriate concentrations for testing.

### 4.4. Oenothera Biennis Hydrophilic Extract (ObHEx) Treatment

NHDF cells were incubated for 24 h with 0.01% (*p*/*v*) ObHEx in complete medium. Subsequently, the cells were washed three times with PBS and treated for an additional 24 h with 0.01% (*p*/*v*) ObHEx in a serum-free medium. They were, then, detached by trypsinization, centrifuged at 500 g for 10 min at 4 °C and washed twice with PBS. Non-treated control cells underwent the same incubations without ObHEx.

### 4.5. Sample Preparation for Proteomic Analysis

Pellets were resuspended in 60µL of Radioimmunoprecipitation assay (RIPA) buffer and lysated by sonication. The cellular lysates protein concentration was quantified using a DC™ Protein Assay Kit (Biorad; #5000112). S-TrapTM micro spin column (Protifi, Hutington, CA, USA) digestion was performed on 50 µg of cell lysates according to the manufacturer’s instructions. Briefly, the samples were reduced with 20 mM tris(2-carboxyethyl)phosphine (TCEP) and alkylated with 50 mM chloracetamide (CAA) for 15 min at room temperature. Aqueous phosphoric acid was then added to a final concentration of 2.5% followed by the addition of an S-Trap binding buffer (90% aqueous methanol, 100 mM TEAB, pH 7.1). The mixtures were then loaded onto S-Trap columns. Five extra washing steps were performed for thorough SDS elimination. Then, the cellular lysates were digested with 2.5 µg of trypsin (Promega) at 47 °C for 1 h. After elution, the peptides were vacuum dried, resuspended in 2% ACN, 0.1% FA and quantified by Nanodrop.

### 4.6. nanoLC-MS/MS Protein Identification and Quantification

A total of 400 ng of each sample was injected on a nanoElute (Bruker Daltonics, Bremen, Germany) high-performance liquid chromatography (HPLC) system coupled to a timsTOF Pro (Bruker Daltonics, Bremen, Germany) mass spectrometer. HPLC separation (Solvent A: 0.1% formic acid in water; Solvent B: 0.1% formic acid in acetonitrile) was carried out at 250 nL/min using a packed emitter column (C18, 25 cm × 75 μm 1.6 μm) (Ion Optics, Fitzroy, Australia) using a gradient elution (2 to 13% solvent B during 41 min; 13 to 20% during 23 min; 20% to 30% during 5 min; 30% to 85% for 5 min, and, finally, 85% for 5 min to wash the column). Mass-spectrometric data were acquired using the data independent analysis parallel accumulation serial fragmentation (diaPASEF) acquisition method. The diaPASEF settings were: mass range from 400 to 1200 Da, mobility ranges from 0.60 to 1.43 1/k0, number of mobility window of 1, cycle time estimate of 1.79 s, mass steps per cycle of 32.

### 4.7. MS Data Processing and Bioinformatics Analysis

Data analysis was performed using DIA-NN software (version 1.8) [44]. A search against the human UniProtKB/Swiss-Prot Homo sapiens database (release February 2021, 20,408 entries) was performed using library free workflow. For this purpose, “FASTA digest for library free search/library generation” and “Deep learning spectra, RTs and IMs prediction” options were checked for precursor ion generation. A maximum of 2 trypsin missed cleavages was allowed and the maximum variable modification was set to 5. Carbamidomethylation (Cys) was set as the fixed modification, whereas protein N-terminal methionine excision, methionine oxidation and N-terminal acetylation were set as variable modifications. The peptide length range was set to 7–30 amino acids, precursor charge range 2–4, precursor m/z range 300–1800, and fragment ion m/z range 200–1800. To search the parent mass and fragment ions, accuracy was inferred automatically by DIA-NN and was set around 13 ppm for each analysis. The false discovery rates (FDRs) at the protein and peptide level were set to 1%. Match between runs was allowed. For the quantification strategy, Robust LC (high precision) was used as advised in the software documentation, whereas default settings were kept for the other algorithm parameters.

Statistical and bioinformatic analysis were performed with Perseus software (version 1.6.15) freely available at www.perseus-framework.org (accessed on 22 June 2021) [45] and R/R Studio (R version 4.1.2 accessed on 5 May 2022, https://cran.r-project.org/bin/windows/base/old/ to access old releases) and RStudio version 2021.09.1 30 (accessed on 12 November 2021). All R statistical analysis was performed using the R stats package. The pg report matrix output by DIA-NN was used and intensities were log2 transformed for statistical analysis. For the statistical comparison, we set four groups, each containing 5 biological replicates. We then filtered the data to keep only proteins with at least 3 valid values in at least one group. Next, the data were imputed to fill missing data points by creating a Gaussian distribution of random numbers with a standard deviation of 33% relative to the standard deviation of the measured values and a 1.8 standard deviation downshift of the mean to simulate the distribution of low signal values. Student’s *t*-test was performed between SEN and CTRL FDR < 0.05, S0 = 0.1 to confirm the presence of markers specific to senescence. Then, in order to investigate if the difference of the effect size between the absence or presence of the treatment is the same for cells where senescence was induced or not, the interaction between both factors (i.e., Induction and Treatment) was investigated using Two-way ANOVA in R. Then, *p*-values obtained for the interaction of both factors were adjusted for multiple testing using the Benjamini–Hochberg [46] method in order to control the False Discovery Rate (FDR). Finally, Tukey HSD post hoc analysis was performed on proteins showing a *q*-value < 0.05. The mass spectrometry proteomics data have been deposited to the ProteomeXchange Consortium via the PRIDE [47] partner repository with the dataset identifier PXD034222.

### 4.8. Senescence-Associated ß-Galactosidase Staining

Senescence-associated ß-galactosidase (SA-ß-gal) activity was evaluated using a Cell Signalling Technology staining kit (#9860). A total of 125′000 NHDF cells/well were seeded into a 6-well plate one day prior to SIPS; whereas, as a control, 25′000 cells/well. After 4 days in normal medium, the cells were incubated or not with 0.01% (*p*/*v*) ObHEx for 48 h. Subsequently, they were washed with PBS and treated with the fixing solution for 15 min. After two washes with PBS, the cells were incubated with the ß-gal staining solution (final pH of 6.0) containing 5-bromo-4-chloro-3-indolyl-β-D-galacto-pyranoside (X-Gal) at 37 °C in a dry incubator for 20 h. Positive cells are blue. The color is due to the cleavage of X-Gal in galactose and 5-Bromo-4-chloro-3-indoxyl (X) by SA-ß-gal. The indoxyl is oxidized to 5,5′-dibromo-4,4′-dichloro-indigo that forms an intense blue precipitate. The percentage of positive cells in the total cells was assessed by counting 100–150 cells in 5 randomly selected images captured by the microscope, for each condition. Cells were counted using ImageJ software. The experiment was performed in triplicate.

### 4.9. Fluorescence-Activated Cell Sorting Analysis

A total of 100′000 NHDF cells/well were seeded into a 6-well plate one day prior to SIPS; whereas, as a control, 5′000 cells/well. After 4 days in normal medium, the control and senescent cells were treated or not with 0.01% (*p*/*v*) ObHEx for 72 h. Then, the cells were incubated in the presence of 5 µg/mL of Hoechst 33342 for 30 min at 37 °C. After trypsinization and centrifugation at 500 g for 2 min, they were resuspended in 200 μL of PBS. Finally, cell fluorescence was measured by a BD LSRFortessa Cell Analyzer. Data were analysed using FlowJo software v10.8.1. The experiment was performed in triplicate.

## 5. Conclusions

The deep senescence-associated global proteome profiling obtained here provides hundreds of proteins deregulated by SIPS that can be exploited by the scientific community to further comprehend senescence and to evaluate the effects of new potential modulators. Moreover, our work proves a promitotic mechanism of action of ObHEx on senescent human dermal fibroblasts: via an increase in mitotic protein expression, it promotes the restoration of the proliferation of senescent cells. Thus, on the basis of these results, we propose ObHEx as a powerful adjuvant against senescence associated with skin aging.

## Data Availability

The mass spectrometry proteomics data have been deposited to the ProteomeXchange Consortium via the PRIDE [47] partner repository with the dataset identifier PXD034222 (Reviewer account details: Username: reviewer_pxd034222@ebi.ac.uk; Password: fK9Pjv90).

## Supplementary Materials

The following supporting information can be downloaded at: https://www.mdpi.com/article/10.3390/ijms232315153/s1.
