# Supplementary material for: Promitotic Action of Oenothera biennis on Senescent Human Dermal Fibroblasts
Source: Int J Mol Sci. 2022 Dec 2;23(23):15153. doi: 10.3390/ijms232315153 (PMC9735661; doi:10.3390/ijms232315153)
Supplement: Supplementary file 1 [file ijms-23-15153-s001.zip › Figure S1.pdf]

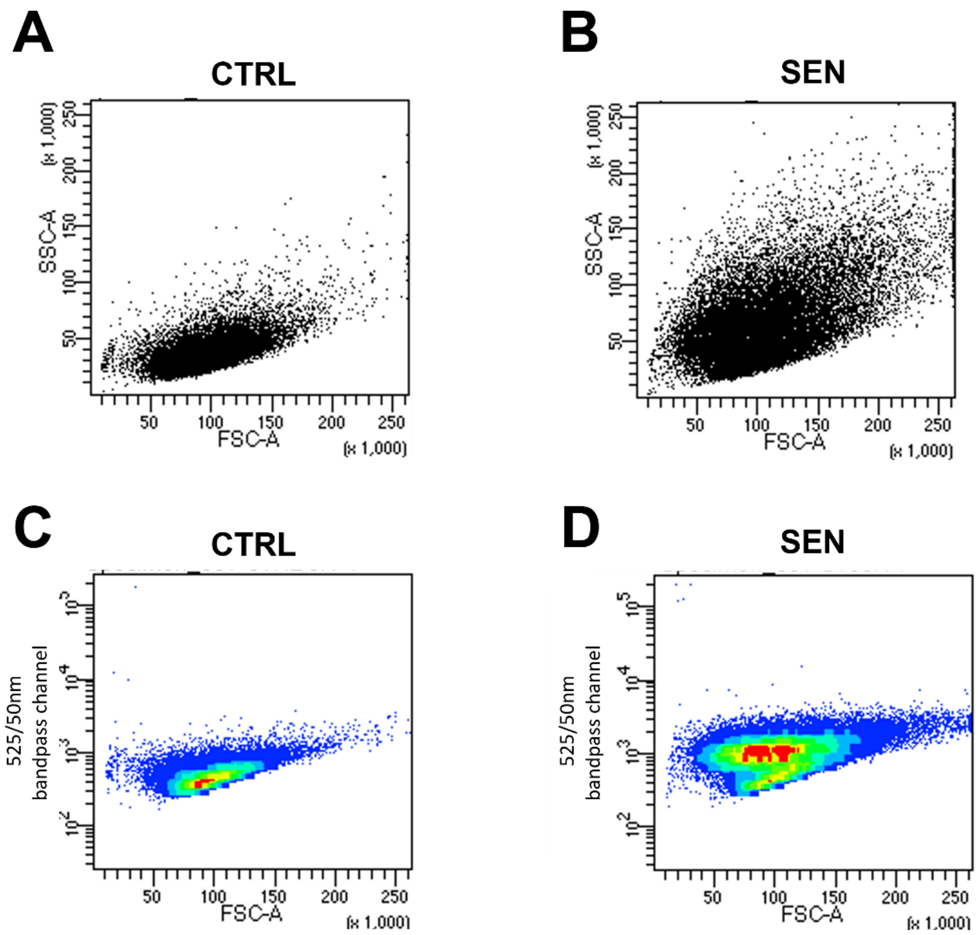

Figure S1. Increase of cytoplasmic granularity and lipofuscin aggresomes in H_2O_2 treated cells. H_2O_2 treated cells (**B**) in comparison with control ones (**A**) showed in FACS (Fluorescence Activated Cell Sorting) analysis an increase of SSC intensity (Side SCatter) that is a measure of cytoplasmatic granularity. It reflects the characteristic increment in lysosomal content of senescent cells. FSC (Forward Scatter) intensity represents the measure of the relative size of the cells. Moreover, in H_2O_2 treated cells a population with high autofluorescence in 525/50nm bandpass channel from the 488nm excitation wavelengh has been detected (**D**) in comparison with the control (**C**). This is a marker of senescent intra-lysosomal accumulation of lipofuscin aggresomes.
