# Supplementary material for: Promitotic Action of Oenothera biennis on Senescent Human Dermal Fibroblasts
Source: Int J Mol Sci. 2022 Dec 2;23(23):15153. doi: 10.3390/ijms232315153 (PMC9735661; doi:10.3390/ijms232315153)
Supplement: Supplementary file 1 [file ijms-23-15153-s001.zip › Table S4. Statistically significant downregulated proteins in senescent NHDF cells after ObHEx treatment.pdf]

Table S4. Statistically significant downregulated proteins in senescent NHDF cells after ObHEx treatment vs untreated senescent NHDF.

Genes	First Protein Description	Diff. (SEN ObHex-SEN Untreated)	adj.p-value
SARS1	Serine--tRNA ligase, cytoplasmic	-0.07218	0.032106
PSMD2	26S proteasome non-ATPase regulatory subunit 2	-0.09294	0.010652
MAP4K4	Mitogen-activated protein kinase kinase kinase kinase 4	-0.0931	0.043178
ASAP2	Arf-GAP with SH3 domain, ANK repeat and PH domain-containing protein 2	-0.1026	0.029708
IQSEC1	IQ motif and SEC7 domain-containing protein 1	-0.1178	0.011567
TP53I3	Quinone oxidoreductase PIG3	-0.1184	0.000432
TAOK3	Serine/threonine-protein kinase TAO3	-0.11934	0.013636
GCLM	Glutamate--cysteine ligase regulatory subunit	-0.1198	0.023221
NPEPL1	Probable aminopeptidase NPEPL1	-0.1267	0.017765
ABHD14B	Protein ABHD14B	-0.12796	0.00735
ITGA2	Integrin alpha-2	-0.13014	0.023285
HSD17B12	Very-long-chain 3-oxoacyl-CoA reductase	-0.13356	0.003779
PLCD1	1-phosphatidylinositol 4,5-bisphosphate phosphodiesterase delta-1	-0.13386	0.008673
PRKAB1	5'-AMP-activated protein kinase subunit beta-1	-0.13852	0.021847
DNAJC16	DnaJ homolog subfamily C member 16	-0.14622	0.010369
SLC12A4	Solute carrier family 12 member 4	-0.15802	0.002591
SIRPA	Tyrosine-protein phosphatase non-receptor type substrate 1	-0.162	0.003137
CHP1	Calcineurin B homologous protein 1	-0.16454	0.018309
SYNJ1	Synaptojanin-1	-0.16664	0.001728
PANK2	Pantothenate kinase 2, mitochondrial	-0.1721	0.004042
PIK3C3	Phosphatidylinositol 3-kinase catalytic subunit type 3	-0.1758	0.015054
CAVIN2	Caveolae-associated protein 2	-0.1783	0.000637
ADAM17	Disintegrin and metalloproteinase domain-containing protein 17	-0.18568	0.007884
NCS1	Neuronal calcium sensor 1	-0.1872	0.002146
ADCY9	Adenylate cyclase type 9	-0.19242	0.000295
LMO7	LIM domain only protein 7	-0.202	1.19E-06

GPX1	Glutathione peroxidase 1	-0.20412	0.009193
TIGAR	Fructose-2,6-bisphosphatase TIGAR	-0.20528	0.001872
MTMR9	Myotubularin-related protein 9	-0.22762	8.82E-05
DGKA	Diacylglycerol kinase alpha	-0.24398	0.000113
PALM	Paralemmin-1	-0.26548	0.01812
PALM2	Paralemmin-2	-0.26738	4.09E-05
LAMC1	Laminin subunit gamma-1	-0.27732	6.83E-07
PRKAR2A;PRKAR2B	cAMP-dependent protein kinase type II-alpha regulatory subunit	-0.28102	2.61E-05
HSPA4L	Heat shock 70 kDa protein 4L	-0.28498	7.41E-05
FDXR	NADPH:adrenodoxin oxidoreductase, mitochondrial	-0.30498	2.5E-06
STK39	STE20/SPS1-related proline-alanine-rich protein kinase	-0.3394	0.000342
CES2	Cocaine esterase	-0.3908	1.01E-05
ASDURF	ASNSD1 upstream open reading frame protein	-0.41014	1.13E-05
FAM117B	Protein FAM117B	-0.4196	0.001711
AKT1S1	Proline-rich AKT1 substrate 1	-0.47006	3.31E-05
ASAP1;ASAP2	Arf-GAP with SH3 domain, ANK repeat and PH domain-containing protein 2	-0.69434	8.12E-05
EPB41L1;EPB41L2;EPB41L3	Band 4.1-like protein 2	-1.04344	6.77E-06
CYB561D2	Cytochrome b561 domain-containing protein 2	-1.0961	6.78E-05
GLRX	Glutaredoxin-1	-1.42864	1.55E-05
C15orf40	UPF0235 protein C15orf40	-2.01816	1.54E-06
