# Supplementary material for: Promitotic Action of Oenothera biennis on Senescent Human Dermal Fibroblasts
Source: Int J Mol Sci. 2022 Dec 2;23(23):15153. doi: 10.3390/ijms232315153 (PMC9735661; doi:10.3390/ijms232315153)
Supplement: Supplementary file 1 [file ijms-23-15153-s001.zip › Table S5. Statistically significant upregulated proteins in senescent NHDF cells after ObHEx treatment.pdf]

Table S5. Statistically significant upregulated proteins in senescent NHDF cells after ObHEx treatment vs untreated senescent NHDF.

Genes	First Protein Description	Diff. (SEN ObHex -SEN Untreated)	adj.p-value
ZNRF1	E3 ubiquitin-protein ligase ZNRF1	1.230648	0.000816
IQGAP3	Ras GTPase-activating-like protein IQGAP3	0.74476	1.16E-07
MCM5	DNA replication licensing factor MCM5	0.68518	2.31E-08
MCM2	DNA replication licensing factor MCM2	0.68264	6.27E-08
CDK1	Cyclin-dependent kinase 1	0.66352	3.65E-05
DHFR	Dihydrofolate reductase	0.64416	0.000118
MCM7	DNA replication licensing factor MCM7	0.63934	1.74E-07
MCM4	DNA replication licensing factor MCM4	0.58212	3.94E-08
EBF1;EBF2;EBF3	Transcription factor COE3	0.57342	2.85E-06
MCM3	DNA replication licensing factor MCM3	0.53704	6.34E-07
PBK	Lymphokine-activated killer T-cell-originated protein kinase	0.51146	0.005872
MCM6	DNA replication licensing factor MCM6	0.50724	2.18E-06
NCAPD2	Condensin complex subunit 1	0.44542	0.000284
TRIP13	Pachytene checkpoint protein 2 homolog	0.43794	6.16E-08
NCAPH	Condensin complex subunit 2	0.4153	0.001812
NCAPG	Condensin complex subunit 3	0.39052	0.000692
COL14A1	Collagen alpha-1(XIV) chain	0.38104	7.09E-07
NUF2	Kinetochore protein Nuf2	0.34564	0.026019
CYP1B1	Cytochrome P450 1B1	0.31314	1.18E-06
CPM	Carboxypeptidase M	0.28808	4.38E-05
KNTC1	Kinetochore-associated protein 1	0.27894	2.07E-05
SMC4	Structural maintenance of chromosomes protein 4	0.25502	1.08E-05
SMC2	Structural maintenance of chromosomes protein 2	0.208	0.001746
ACO1	Cytoplasmic aconitate hydratase	0.17734	0.000311
LAMB1	Laminin subunit beta-1	0.16292	1.98E-05
